# Association Between System Reach and Exposure to Interventions and Characteristics of Mobile Female Sex Workers in Four High HIV Prevalence States in India

**DOI:** 10.5539/gjhs.v7n4p83

**Published:** 2014-12-31

**Authors:** Varun Sharma, Niranjan Saggurti, Shalini Bharat

**Affiliations:** 1 School of Health Systems Studies, Tata Institute of Social Sciences, Mumbai, India; 2 HIV/AIDS Program, Population council, New Delhi, India

**Keywords:** exposure to interventions, HIV, mobile female sex workers, systems’ agents, India

## Abstract

Mobility among Female Sex Workers (FSWs) interrupts their demand for, and utilization of, health services under any intervention. Various strategic interventions are meant to provide access to care and reduce the incidence of HIV and other STIs among FSWs. This paper applies a bivariate probit regression analysis to explain the probability of mobile FSWs being reached by the system and being exposed to interventions jointly with a wide variety of characteristics of mobile FSWs in India. The data used are based on a cross-section survey among 5,498 mobile FSWs in 22 districts of four high HIV prevalence states in southern India. A majority of mobile FSWs (59%) were street-based and about 70 percent of them were members of SW organization and nearly half (46%) were highly mobile. The majority of them (90%) had been contacted by outreach workers from any system in the last two years in their current location and 94 percent were exposed to interventions in terms of getting free or subsidized condoms. Bivariate probit analysis revealed that comprehensive interventions are able to reach more vulnerable mobile FSWs effectively, e.g. new entrants, highly mobile, reported STIs, tested for HIV ever and serving a high volume of clients. The results complement the efforts of government and other agencies in response to HIV. However, the results highlight that specific issues related to various subgroups of this highly vulnerable population remain unaddressed calling for tailoring the response to the specific needs of the sub-groups.

## 1. Introduction

Globally, female sex workers (FSWs) are most vulnerable to infection with HIV (Chattopadhyay & McKaig, 2004; Morris, Morris, & Ferguson, 2009). High prevalence of HIV and other STIs among them is the result of their inconsistent or no use of condom with clients, particularly non-paying clients (Chattopadhyay & McKaig, 2004; Dandona et al., 2005; Ministry of Public Health and Sanitation, 2010; Morris et al., 2009; Munoz et al., 2010; Saggurti et al., 2011), frequency and number of clients (Morris et al., 2009), unprotected anal sexual practice (Alexander et al., 2014; Dandona et al., 2005; Ministry of Public Health and Sanitation, 2010) and substance use before or during sexual intercourse (Saggurti et al., 2012). They are also ‘difficult to trace population’ given the nature of their work and associated stigma (Ministry of Public Health and Sanitation, 2010). Poor work conditions and environment (violence and exploitation) (Dandona et al., 2005), low economic condition (Dandona et al., 2006; Saggurti et al., 2011), less control over their earnings (Ngo et al., 2007), control of madams and pimps and threat of police raids, societal disapproval, and stigma and discrimination, poor access to knowledge and awareness related to sexually transmitted infections (STIs) and HIV (Rakhi Dandona et al., 2006) and low access to health facilities, further accentuate their vulnerability to HIV and STIs. Gender, income and power inequalities intersect to restrict and limit the capability of FSWs to negotiate safer sexual practices with clients (Bharat, Mahapatra, Roy, & Saggurti, 2013; Misra, Mahal, & Shah, 2000; Ngo et al., 2007). Mobility of FSWs, driven by work uncertainties, further enhances their risk to HIV and interrupts access to health care and services under any intervention (Dandona et al., 2006; Ministry of Public Health and Sanitation, 2010; Ramesh, Ganju, Mahapatra, Mishra, & Saggurti, 2012; Reed, Gupta, Biradavolu, & Blankenship, 2012; Saggurti et al., 2012). Research studies suggest that FSWs’ access to care and services are generally not fully addressed, accepted, and made accessible or affordable due to stigmatization and marginalization by the health and programme staff. Further, unsupportive legal arrangements for them lead to hidden and unsafe sexual practices and limiting their access to services (Dandona et al., 2006; Misra et al., 2000).

Strategic targeted interventions [e.g. Community empowerment and mobilization, drop-in-centres, community committees, advisory groups and social support systems] are found to reduce HIV risk and vulnerability among FSWs considerably (Arora, Nagelkerke, Moineddin, Bhattacharya, & Jha, 2013; Bhave et al., 1995; Campos et al., 2013; Gurung et al., 2011; Ministry of Public Health and Sanitation, 2010; NACO, 2012; WHO., UNFPA., UNAIDS., NSWP., & WB., 2013). These targeted interventions (TIs) typically aim at primary HIV prevention and deliver treatment for sexually transmitted infections (STIs), condom provision, behaviour change communication, creation of a supportive environment using community involvement and participation (peer-based educators) and linkage to treatment and support services (Arora et al., 2013; Basu et al., 2004; Reza-Paul et al., 2012). As a key component of HIV prevention programme, Peer Educators (PEs) and Outreach Workers (ORWs) are found to play a vital role in preventing high risk sexual behaviour among FSWs, reduce substance use, increase participation in sex workers collectives and mobilizing them, increase participation in risk reduction behaviors such as correct and consistent use of condom with all clients, demand for health care services, screening and seeking treatment of any STI symptoms and voluntary HIV testing (Arora et al., 2013; Basu et al., 2004; Campos et al., 2013; Reza-Paul et al., 2012). Coverage and effective delivery of services of intervention are dependent on PE and OR activities largely. Sustainability and adaptability of such interventions, on the other hand, will rely on the degree to which they achieve their set outcome of change in risky sexual behaviour (Gurung et al., 2011).

HIV intervention programmes among FSWs have been implemented effectively in Brazil (Pegacao, 1991), Thailand and Zaire (Schoepf, 1993). In Indian context, FSW interventions have expanded impressively, mostly in the high HIV burden states [Maharashtra, Tamil Nadu, Andhra Pradesh and Karnataka] and moderately in other states. These programmes range from structural intervention, community-led-interventions, advocacy and enabling environment, mapping exercise, condom programmes, clinical services, outreach services, behaviour change communication, management of services to testing, care, support and treatment (Basu et al., 2004). These TIs and Peer-based programmes are designed to reach the maximum number of FSWs to provide preventive and curative services to them (BMGF, 2008; Gurung et al., 2011). Department of AIDS Control (DAC; previously called National AIDS Control Organisation- NACO), the nodal agency of the Government of India has supported sex worker interventions in many high HIV prevalence districts since the mid 1990s through its National AIDS Control Programme (NACP). NACP activities and targeted interventions have substantially increased over the years. Programme data show that comprehensive TIs tend to bring most of the FSWs at a common platform and expose them to interventions and link them to the system. It is estimated that there are about 8.68 lakh^1^ FSWs in India, scattered in different states of the country. Out of that, about 7.34 lakh FSWs (84.5%) are being covered through 508 TI projects (NACO, 2012). Apart from DAC, the reach of other international agencies [UNAIDS, UNDP, UNICEF and GFATM etc.] in the area of prevention of HIV is also appreciable. The percentage coverage of estimated number of FSWs in India through different TIs has tremendously increased from 53.4 per cent in 2009-10 to 84.5 per cent in 2012-13 (NACO, 2012). Likewise, through rigorous efforts under NACP, significant achievements have been made, in terms of availability of condoms and increase in the awareness about condom use in HIV/AIDS prevention. NACP has successfully implemented and scaled up the ‘Condom Social Marketing Programme’ from 194 districts in Phase–I (2008-2009) and to 294 districts in Phase–II (2009-2010) (NACO, 2012). Alike, Bill & Melinda Gates Foundation’s (BMGF) Avahan program had spent US$250 million since 2004 for expanding and scaling up activities with government bodies to reach the maximum number of the most at risk population (BMGF, 2008). The consistent efforts of the government of India and other international agencies are visible in terms of significant reduction in HIV and STIs prevalence among FSWs, increase in correct and consistent use of condom and increase in their ability to negotiate for safer sexual practices in the recent years (Arora et al., 2013; Bharat et al., 2013; Gurung et al., 2011).

This paper attempts to relate targeted interventions [the system reach and exposure to interventions] with characteristics of mobile FSWs in India. Mobile FSWs is difficult to reach sub-group among FSWs. Due to their high mobility and other socio-demographic traits, they are difficult to trace and to be contacted by ORWs and thus limiting their access to services under any intervention.

In this paper, system reach is defined in terms of contacts by ORWs or PEs and exposure to interventions is defined in terms of access to free or socially marketed condoms (SMC). ORWs/PEs are considered as agents of the system. Here, system connotes to the structural national response to HIV under NACP and/or initiatives of other international agencies in response to HIV. There is a close interdependence between contacts by ORWs and access to free/SMC, as distributing free or SMC to SWs is one among other tasks assigned to ORWs under various interventions. Thus, it is assumed that there are some correlations between system reach and exposure to interventions. Taking into account this correlation, the bivariate probit regression analysis is used to explain the probabilities of mobile SW being reached by the system and exposed to intervention jointly with a range of characteristics of them in India. The bivariate probit regression model is having the advantage of taking into account the correlation between the error terms of two equations analyzed, i.e. being reached by the system and being exposed to interventions.

## 2. Method

### 2.1 Study Design

A mixed methods study involving in-depth interviews and survey with mobile FSWs was used. The study areas were identified and characterized using primarily qualitative data, followed by quantitative survey.

### 2.2 Qualitative Interviews

Qualitative interviews were carried out with local key informants, including FSWs, selected mobile FSWs and groups of SWs. Qualitative interviews were conducted in 2007-08 prior to quantitative survey, in four high HIV prevalence states [Maharashtra, Andhra Pradesh, Karnataka and Tamil Nadu] covering 22 districts and different FSWs [brothel based, street based and home based]. A total 367 in-depth interviews with mobile FSWs were conducted in the selected study sites.

### 2.3 Quantitative Survey

A quantitative survey was conducted among FSWs aged 18 years and older and who had moved at least two places for sex work [one of which included a move across districts] in the last two years.

### 2.4 Study Sites

For the quantitative survey, data were collected from a cross-section survey among mobile FSWs in 22 districts from four high HIV prevalence states in southern India (Andhra Pradesh, Karnataka, Maharashtra, & Tamil Nadu). The data were collected from September 2007 to July 2008.

### 2.5 Sampling Procedure and Sample Size

For the quantitative survey, a two-stage sampling procedure was used to select FSWs from both brothel and non-brothel sites. A detailed mapping of the large and small solicitation sites was done and the list was used to define and select cluster sites, which included both small and large area and covering approximately 500 FSWs in each cluster. Three clusters were randomly selected and FSWs were systematically sampled. The sample size was determined using an estimated proportion of 30% non-condom use, an assumed difference of 3% increase in the proportion with every unit increase in degree of mobility, a confidence level of 95% and power of 80%. An analytical sample of 5,498 FSWs was drawn. Detailed methodology and data collection procedure is discussed elsewhere (Saggurti et al., 2012). Ethical approval for the study was obtained from the institutional review boards (IRBs) of the Population Council and the University of Manitoba, Canada. In this paper responses of 5,487 mobile FSWs, with no missing values for the areas of enquiry were analyzed.

### 2.6 Data Collection Tool

A structured questionnaire with detailed, in-depth guidelines was used to collect information from mobile FSWs regarding their demographic and socio-economic status, sexual behaviour with different clients, access, availability and use of condom, STI symptoms, mobility nature and pattern, system reach, media exposure, substance use, likelihood to move further and HIV awareness and self-perceived risk.

The analysis in this paper is limited to a few questions from this tool. The dependent variables used in this paper are listed in section 7 and section 19 of the interview schedule. Based on those questions dependent variables are framed as follows:

#### 2.6.1 System Reach

The respondents were asked questions regarding contact by outreach workers and use of any health facility in the past two years at the current location. Interviewees were asked *“Has anyone from the following systems ever reached you in the past two years: Outreach worker Government, Outreach worker from NGOs: other agencies, Outreach worker from NGOs: Avahan?”* If the respondent was contacted by ORW from any system in the last two years, it was scored **1**. ORWs were described as systems’ agent.

#### 2.6.2 Exposure to Interventions

Exposure to interventions was measured by asking the respondents, *“Are condoms available free-of-cost here? Or do you get subsidized condoms here? (Socially marketed condoms)?”* If the respondent stated receiving either, free or socially marketed condoms it was scored as **1**.

### 2.7 Analytical Strategy

A bivariate probit regression analysis is employed to assess the association between system reach and exposure to interventions with characteristics of mobile female sex workers. The log-likelihood function is derived and the parameters are estimated by Maximum Likelihood estimation. A bivariate probit model assumes that the “independent, identically distributed” errors are correlated (Greene, 2003; Jones, 2007). Quantitative analysis was carried out using STATA 12.0 and qualitative analysis was carried out using ATLAS ti.

#### 2.7.1 Estimation of Probability of Being Contacted by Systems’ Agent and Exposed to the Intervention

Coefficients [β] of bivariate probit model are not measured in natural units and thus they can only give a qualitative interpretation. However, the marginal and average effect gives the impact of a change of one of the explanatory variables on the marginal probability of each outcome, for example, the probability of a mobile FSW being contacted by any ORW, or the probability of her being exposed to interventions. Secondly, it is possible to calculate the marginal effect of an explanatory variable on the joint probability of each of the four outcome combinations. Likewise, it is possible to calculate the marginal effects of the explanatory variables on conditional probabilities, for example the probability that a FSW being exposed to any intervention, given that she was reached by the ORW.

### 2.8 Variables Description

Dataset detailed in methods section provides the necessary variables for the study. The necessary variables used in the regression are *‘System reach’* and *‘Exposure to interventions’*, *‘Age’*, *‘Typology sex worker’*, *‘Educational status’*, *‘Duration of sex work’*, *‘Living arrangements’*, *‘Membership of any organization’*, *‘Frequency of movements in the last two years’*, *‘Ever tested for HIV’*, *‘Frequency of clients in the last week worked’* and *‘STI symptoms’*.

## 3. Results

Table 1 shows the summary statistics on the variables used in the analysis. The independent variables in the equation are young FSW, typology of sex workers, educational status of FSW, duration in sex work, ever tested for HIV infection, living arrangements, membership of any organization, frequency of mobility, any symptom of STI in the last six months and clients served in the last week worked.

**Table 1:** Summary statistics of variables used

Description of variables	Frequency [n=5498]	Percentage of n
System reach	4,992	90.8
Exposure to interventions	5,169	94.0
Typology of sex workers		
Street based FSWs (SBS)	3,221	58.6
Home based FSWs (HBS)	1,095	19.9
Brothel based FSWs (BBS)	1,182	21.5
Educational status		
Non-literate	1,893	34.4
Up to primary	1,817	33.0
Above primary	1,788	32.5
Duration into sex work		
New entrants [FSWs in sex work less than equal to 2 years]	1,104	20.1
FSWs who have ever been tested for HIV infection	4,475	81.4
Living arrangements^a^		
FSWs living alone	667	12.1
FSWs living with family or any other relative	3,568	64.9
FSWs living with other co-sex workers or pimps	1,252	22.8
FSWs member of any organization	3,917	71.2
Highly mobile FSWs [Had moved more than 4 places in the last two years]	2,499	45.5
Young FSWs [FSWs less than equal to 25 years]	1,321	24.0
Had any STI symptom in the last six months	3,922	71.3
Serving high volume of clients in the last week worked [More than 10 clients in the last week worked]	3,445	62.7

a 11 respondents did not answer about their living arrangement.

Of the sampled population [n= 5498], 24 percent were in the age group of less than or equal to 25 years, i.e. every fourth FSWs was categorized as ‘young’ and 34 percent were illiterate. A majority of mobile SWs (59%) were street-based. The percentage of new entrants into sex work was 20 percent. A high percentage (81%) had ever been tested for HIV infection. The percentage of those who were living alone was 12 percent and around 65 percent were living with family or any other relative. A relatively low percentage (23%) was residing with co-sex workers or pimps. Around 70 percent of FSWs were members of any organization and nearly half (46%) were highly mobile *i.e*. had moved more than four places for sex work in the last two years. A high percentage (71%) had reported any STI symptom in the last six months. A relatively high percentage of FSWs (63%) was serving a high volume of clients *i.e*. more than 10 clients in the last week worked. Table 2 shows that 90 percent mobile FSWs had been contacted by ORW from any system in the last two years in the current place and 94 percent were exposed to interventions in terms of getting free or subsidized condoms.

**Table 2:** Characteristics of mobile SWs not reached by the system and not exposed to interventions

Characteristics	Those who were not reached by the system [n=506]		Those who were not exposed to any intervention [n=329]	
Age categories				
15-19	1	(0.2)	4	(1.22)
20-24	60	(11.86)	50	(15.2)
25-29	210	(41.5)	149	(45.29)
30-34	129	(25.49)	75	(22.8)
35-39	59	(11.66)	33	(10.03)
40+	47	(9.29)	18	(5.47)
Educational status				
Non-literate	174	(34.39)	142	(43.16)
Primary	160	(31.62)	100	(30.4)
Above Primary	172	(33.99)	87	(26.44)
Typology of sex workers				
BBS	50	(9.88)	72	(21.88)
SBS	404	(79.84)	229	(69.6)
HBS	52	(10.28)	28	(8.51)
Mobility				
Less mobile [<=4 places]	310	(61.26)	236	(71.73)
High mobile [>4 places]	196	(38.74)	93	(28.27)
Membership of any organization				
No membership	441	(87.15)	295	(89.67)
Membership	65	(12.85)	34	(10.33)
Caste status^a^				
Schedule caste	79	(21.01)	50	(29.94)
Schedule tribe	38	(10.11)	26	(15.57)
Other backward class	166	(44.15)	63	(37.72)
Others	93	(24.73)	28	(16.77)
Duration into sex work				
Others [>2 years]	405	(80.04)	267	(81.16)
New entrants [<=2 years]	101	(19.96)	62	(18.84)

a Few SWs did not respond to their caste status, figures in bracket are percentage, BBS: Brothel based sex workers, SBS: Street based sex workers, HBS: Home based sex workers

Around 10 percent of all (9.6%) were not reached by systems’ agents and 6% were not exposed to any intervention. Among those who were not reached by systems’ agents and were not exposed to any intervention, the majority of them were under the age of 30 years and were street based sex workers. Table 2 shows the characteristics of mobile SWs who were not reached by the system and exposed to interventions.

The bivariate probit model technique is used to estimate the probability of a mobile SW being *“approached by systems’ agent”* together with the probability of being *“exposed to interventions”* with characteristics of FSWs as explanatory variables.

From the hypothesis testing of bivariate probit, the result is significantly different from zero of rho [ρ] where rho equals to [0.46]. This means that there is some covariance of error terms between the probability of being approached by systems’ agent and exposed to interventions. Table 3 shows the results based on bivariate probit regression.

**Table 3:** Bivariate probit regression results

Number of observations = 5487 wald chi2 (24)= 1074.54					

log likelihood ratio= -2005.4115			prob>chi2 = 0.000		

Variables	Coefficient	Robust Std. Err.	z	p>|z|	[95% CI]
**System reach**						
Young FSW *[Ref: Age>25 years]*	0.016	0.072	0.230	0.821	[-0.124 ― 0.157]
Typology of SWs *[Ref: BBS]*					
SBS	-0.729***	0.096	-7.630	0.000	[-0.916 ― -0.542]
HBS	-0.320**	0.124	-2.570	0.010	[-0.563 ― -0.076]
Educational status *[Ref: Non-literate]*					
Up to primary	0.020	0.072	0.280	0.779	[-0.121 ― 0.161]
Above primary	-0.051	0.070	-0.720	0.473	[-0.189 ― 0.087]
New entrants *[Ref: Duration >2 years]*	0.192**	0.078	2.460	0.014	[0.039 ― 0.345]
Ever tested for HIV *[Ref: Never tested for HIV]*	0.213**	0.069	3.110	0.002	[0.079 ― 0.347]
Living arrangements *[Ref: Living with other sex workers/pimp]*					
Living alone	-0.192*	0.106	-1.820	0.069	[-0.400 ― 0.015]
Living with family or other relatives	0.032	0.076	0.420	0.672	[-0.116 ― 0.180]
Member of any organization *[Ref: No membership]*	1.565***	0.065	24.250	0.000	[1.439 ― 1.692]
High mobility *[Ref: Less mobile]*	0.143**	0.061	2.350	0.019	[0.024 ― 0.263]
Had STI symptoms *[Ref: Never had STI symptoms]*	0.314***	0.060	5.230	0.000	[0.196 ― 0.432]
High inflow of clients *[Ref: Low inflow of clients]*	0.282***	0.063	4.460	0.000	[0.158 ― 0.407]
Constant	0.509	0.143	3.570	0.000	[0.230 ― 0.789]
**Exposure to interventions**						
Young FSW *[Ref: age>25 years]*	-0.011	0.079	-0.140	0.889	[-0.167 ― 0.144]
Typology of SWs *[Ref: BBS]*					
SBS	-0.335**	0.096	-3.480	0.001	[-0.523 ― -0.146]
HBS	-0.017	0.134	-0.130	0.900	[-0.280 ― 0.246]
Educational status *[Ref: Non-literate]*					
Up to primary	0.163**	0.077	2.130	0.033	[0.013 ― 0.314]
Above primary	0.129	0.084	1.550	0.122	[-0.035 ― 0.293]
New entrants *[Ref: Duration in sex work >2 years]*	0.327***	0.094	3.490	0.000	[0.143 ― 0.511]
Ever tested for HIV *[Ref: Never tested for HIV]*	0.354***	0.081	4.380	0.000	[0.195 ― 0.512]
Living arrangements *[Ref: Living with other sex workers/pimps]*					
Living alone	-0.054	0.105	-0.510	0.608	[-0.260 ― 0.152]
Living with family or other relatives	0.487***	0.083	5.850	0.000	[0.324 ― 0.650]
Membership of organization *[Ref: No membership]*	1.411***	0.080	17.690	0.000	[1.255 ― 1.568]
High mobility^a^ *[Ref: Low mobility]*	0.302***	0.073	4.120	0.000	[0.158 ― 0.445]
Had STI symptoms *[Ref: Never had STI symptoms]*	-0.096	0.072	-1.330	0.182	[-0.238 ― 0.045]
High inflow of clients^b^ *[Ref: Low inflow of clients]*	-0.093	0.078	-1.190	0.234	[-0.246 ― 0.060]
Constant	0.498	0.137	3.630	0.000	[0.229 ― 0.768]

/athrho	0.497	0.051	9.810	0.000	[0.398 ― 0.597]
Rho (ρ)	0.460	0.040	0.378	0.535		

Wald test of rho=0: chi2(1) = 96.284 Prob > chi2 = 0.000					

*** Sig @ 99%,

** Sig @ 95%,

* Sig @ 90%, BBS: Brothel based sex workers; SBS: Street based sex workers; HBS: Home based sex workers; CI: Confidence interval;

a High mobility: those who had moved to more than 4 places in the last two years for sex;

b High inflow of clients: those who had served more than 10 clients in the last week of work.

The equation for *‘system reach’* shows that compared to brothel based mobile FSWs, street-based and home-based FSWs have a less probability to be contacted by any ORW. New entrants, members of any organization, those who have reported any symptom of STI in the past six months, those who were ever tested for HIV and those who are highly mobile [as defined in the paper], are more likely to be reached by system agents in the past two years at the current place of location. The probability of those living alone, being contacted by ORW is significantly low.

Likewise, the equation for *‘exposed to intervention’* shows that compared to brothel based FSWs, those who are street based, are less likely to be exposed to interventions and hence less likely to be getting the benefits of interventions. However, no significant association was found between home-based FSWs and probability of being exposed to interventions. Similarly, those who are new entrants, educated up to primary level, ever tested for HIV, living with family or any relative, member of any organization [SHG, CBO, NGO or sex worker collectives] and highly mobile are more likely to be exposed in terms of getting free condoms or socially marketed condoms.

The new information provided by the bivariate probit model is the estimate of rho [ρ] the correlation coefficient for the two error terms. The estimate is 0.46 and the chi-square test of 96.284 shows that this estimate is significantly different from zero. This plausible result indicates that unobservable factors [factors that are not included in the model] are positively related to both outcome variables.

Conditional probability analysis after bivariate probit regression [Table 4] shows that the probability of being approached by systems’ agent together with being exposed to interventions (p11) is [87.7%]; the probability of being approached by systems’ agent, but not being exposed to interventions (p10) is [3.1%]; the probability of being exposed to interventions, but not contacted by systems’ agent (p01) is [6.3%] and probability of being ignorant *i.e*. not approached by systems’ agent and no exposure to interventions (p00) is [2.8%]. It could be implied that in hundred FSWs, there are only two to three FSWs who are not reached by ORW of any system and are unexposed to interventions.

**Table 4:** Outcome variables and predicted probabilities from bivariate probit regression

Variable	Obs.	Mean	Std. Dev.	Min	Max
System reach	5498	0.908	0.289	0.0000	1.0000
Exposure to intervention	5498	0.940	0.237	0.0000	1.0000
Predicted probability of system reach	5487	0.909	0.136	0.3747	0.9995
Predicted probability of exposure to interventions	5487	0.941	0.094	0.4639	0.9998
Joint predicted probability of system reach with exposure to the intervention	5487	0.877	0.168	0.3232	0.9991
Joint predicted probability of system reach and not exposed to interventions	5487	0.031	0.048	0.0002	0.2927
Joint predicted probability of no system reach and exposure to interventions	5487	0.063	0.093	0.0005	0.4235
Joint predicted probability of no system reach and no exposure to interventions	5487	0.028	0.051	0.0000	0.3068

The marginal probabilities that y1 (system reach) = 1 and y2 (exposure to interventions) = 1 are, 0.909 and 0.941, respectively, very close to the sample frequencies (0.908 and 0.940 respectively).

## 4. Discussion

System reach, accessibility and availability of condoms are important aspects of HIV prevention programme for FSWs (Kumar et al., 2011; Munoz et al., 2010). Availability and accessibility of condoms are important catalysts for promoting safer sexual practices among them with the help of ORWs from different systems. This paper documents the probability of mobile FSW being contacted by ORWs and FSW receiving free or social marketed condoms with the characteristics of mobile FSWs in four high HIV prevalence states in India.

Studies have documented that the reach of the system and access to free or socially marketed condoms is significantly low among street based FSWs as compared with brothel based FSWs (Kumar et al., 2006). The finding of this study is in-line with earlier studies. Considering a large number of street based FSWs in India, this finding is relevant from a policy perspective and re-designing the targeted interventions for FSWs. Likewise, their inability to access free or socially marketed condoms or distance from systems’ agents increases their vulnerability to various STIs and HIV infection (Kumar et al., 2006). In a study based in Andhra Pradesh, Kumar et al (2006) found that no access to condom was significantly higher among street based FSWs. The same study reflected that condom requirements were not met for three-fifths of the FSWs (Kumar et al., 2006).

In the paper, SWs, those who are into sex work for less than or equal to two years are defined as new entrants in the sex work. They are more likely to be vulnerable to HIV and other STIs because of being new to the sex work practices and their inability to insist on condom use with clients (Chaudhury, Sutapall, & Latheef., 2011). The results of this paper, however, show that new entrants are more likely to be contacted by system agents and more likely to be getting free or socially marketed condoms. This finding highlights the comprehensive, targeted intervention by different systems involved in response to HIV. The result is in line with the recent study, which also reflected that coverage of the intervention has significantly increased among new entrants in the sex work (Mainkar et al., 2011). However, many new entrants are likely to be under the age of 18 which were not covered under this study. Thus, system reach and intervention exposure among this group is unexplored.

FSWs, those who had ever been tested for HIV are more likely to be contacted by ORWs and are more likely to be getting free condoms. Studies reported that there was a significant increase in the proportion of FSWs exposed to the programme that had undergone an HIV test (Tran et al., 2014). Compared to those who are residing with other co-sex workers the reach of the system is limited among those who are staying alone.

Membership of any organization [SHG, CBO, NGO or sex worker collectives] is another important predictor of probability of a FSW being approached by the system and exposed to the intervention (Erausquin, Biradavolu, Reed, Burroway, & Blankenship, 2012; Kumar et al., 2006). This finding highlights that the collectivism among FSWs or membership of any SHG or CBO may lead to better access to services and more negotiation power and ultimately correct and consistent condom use (Chakravarthy, Joseph, Pelto, & Kovvali, 2012; Erausquin et al., 2012; Kumar et al., 2006). It was also observed that the FSWs who are serving more clients are more likely to be contacted by system agents. A high volume of clients puts them at higher risk of HIV and other STIs. The study found that system reach is effectively high among those who are serving more clients. A study based in Bangalore^2^, India also highlighted that the reach of the system and exposure has increased among new and busier FSWs. These findings are important considering the macro level objective of reducing HIV prevalence in the targeted population.

Likewise, the probability of being approached by any system among those who had reported any STI in the last six months is high. The results are in consensus with the recent studies which also highlighted that the cumulative efforts of government and other agencies are visible in terms of uptake of the programmatic intervention and services (Kokku, Mahapatra, Tucker, Saggurti, & Prabhakar, 2014).

The analysis shows that highly mobile FSWs are more likely to be contacted by systems’ agents and accessing free or socially marketed condoms. These findings revalidate the reach of programmes to the difficult to cover FSWs in the recent years and reiterate the success of the interventions targeting them. However, recent literature related to mobile FSWs in India and elsewhere, showed that various characteristics of mobile FSWs hinder the correct and consistent use of condom and limits their ability to negotiate condom use and access health care (Richter, 2013). There is a need to explore this association in detail as the excerpts from the qualitative interviews of mobile FSWs also suggest that mobile FSWs had access to free condoms from various organizations or TIs of various international agencies [Saheli, Mukta project and PSI etc.] at the locations they visited while being mobile. Some mobile brothel based sex workers stock up condoms for the entire duration of their travel as suggested by the following quote:

*“We get Nirodh (Condoms) from Myrada organization. They provide us a box of Nirodh, which holds 500 condoms. On the off chance that I go outside for sex work, I carry that alongside me. Till Nirodh gets over I do sex work, then I return”*. [Brothel based mobile FSW, aged 20 years, Bellary District, Karnataka].

In other words, a majority of mobile FSWs continues to be served by the system during their travels. The most plausible reason could be that the mobile FSWs are not entirely an independent category and at least some of them are brothel based or home based who take up contracts to serve out of town clients through agents. Not only do they get free or socially marketed condoms, but also information and awareness regarding HIV and safer sexual practices through interventions. Analysis of quantitative data^3^ also revealed that out of total 2,499 highly mobile FSWs 12 percent were brothel based, 65 percent were street based and rest 23 percent were home based. Among brothel based, highly mobile SWs, nearly 99 percent were contacted by systems’ agents. Hence, the system reaches to these FSWs as brothel based sex workers. The percentage of those not contacted by systems’ agent among street based and home based sex workers were 10.3 percent and 4.3 percent respectively. Moreover, 10 percent of highly mobile FSWs were under contracts with madams and pimps and a majority of them (55%) were brothel based. As brothel based SWs, they get information about HIV, AIDS, and safer sexual practices that is suggested by the following quote:

*“Volunteers from one organization used to pay a visit at Hanuman Tekadi [Bhiwandi, Thane]. They disseminate information about HIV and AIDS and inform us that when you have intercourse you ought to essentially utilize condom. In Budhwar Peth [Pune], individuals from Kayakalp organization were indicating pictorials of a woman who had HIV infection. Her life was ruined, she had not utilized condom while engaging in sexual relations. Hence, they used to spread the words that you will need to stop AIDS by utilizing condom. Condoms are available free of charge. They ought to be utilized by clients. I likewise dependably utilize condom. If some individual has AIDS, he ought to consume good sustenance, he ought not to drink liquor. People in his encompassing ought to act with him with affection. They ought to converse with him smilingly and with affection”*. [Brothel based mobile FSW, aged 30 years, Pune district, Maharashtra]

Moreover, mobile FSWs are also aware about STIs. They are aware of the source of treatment and precautions while having STIs as suggested by following quote:

*“We go to a local N.G.O ’Vimochana’ when we have health problems. We do not indulge in sex work when there is white discharge (STI symptom) and when we are having periods (menstruating). We know about HIV/AIDS. HIV virus spreads through unsafe sex, blood transfusion, etc., so we care for that and do not practice sex work without a condom”*. [Mobile FSW, aged 30 years, Vijayawada district, Andhra Pradesh]

Likewise, from quantitative data, mostly brothel based, highly mobile SWs (99.3%) were accessing free or socially marketed condoms. In contrast, 5% and 3% street and home based SWs respectively, were not able to access condoms from any source of intervention. Hence, the needs of those who are street/Dhaba based FSWs are not adequately met as suggested by the following quote:

*“…. I am not using condoms. However, some clients use to bring it. Some use to have sex without condoms… I do not know about the disease (HIV & AIDS), how does it spread and what are the symptoms”*. [Street/Dhaba based FSW, aged 28 years, Ahmednagar district, Maharashtra]

Besides, the complexities arise to define these FSWs in any one designated category as suggested by the following quote:

*“I came back from Rajahmundry [Andhra Pradesh], stayed for 10 days, and then went to a brothel house in Gudivada where I stayed for 20 days… I do not practice sex work at home. Nobody knows that I am a sex worker… When we are in a brothel we have no problems at all. If anybody refuses to use a condom then the brothel owners themselves do not allow such people”*. [Mobile FSW, aged 30 years, Vijayawada district, Andhra Pradesh]

In-depth interviews additionally revealed that these movements of FSWs were noticed within the city and outside the city, district and state territory in a frequent manner. Hence, it can be argued that in recent years, high mobility of FSWs may not be a barrier till the time the programmes directed to them are comprehensive in terms of geographical coverage and service provisioning. In this context, ORWs under various interventions play a significant role. ORWs/PEs are responsible for providing information on HIV/STIs and promoting condom use among colleagues/peers, which ultimately results in building peer pressure for behaviour change. They can also distribute condoms, lubricants, needles and syringes. They also provide basic data for monitoring the project. ORW/PE identifies new FSW as well as maintain regular contact with her own network of FSWs [PE to FSW ratio is set at 1:60 – one PE for 60 FSWs; NACP-III]. This might involve contacts on a weekly or bi-weekly basis within any given month. Likewise, under the Avahan India AIDS initiative, implementation was assigned to nine lead NGOs who ran the programs for high risk groups and clients in the six target states. These large NGOs sub-granted to about 134 grassroots NGOs, which then worked with over 7,000 peer educators and outreach workers who became the face of the program. Under Avahan, PEs/ORWs promoted condom use and attendance at clinics, and encouraged follow-up and partner treatment. Twenty four months into implementation, 83 percent of targeted high risk population members had been contacted by a peer educator at least once. Thus, it can be argued that despite high mobility SWs were contacted by system agents and can be attributed to comprehensiveness of different programs.

However, the implications of these movements of FSWs on the programmatic interventions, particularly on impact assessment studies in a geographical area cannot be discounted. Those who move out may be difficult to trace and hence may be lost in the tracking mechanism.

The findings of the paper estimate the probability of system and benefits of programs reaching to mobile FSWs and report that programmes for the prevention and care among mobile FSWs are able to reach most of them. Thus, not all mobile FSWs are missed out. Those who are brothel based are covered even while being mobile. However, there is a need to develop (and/or to tailor the existing programmes) to reach and address the particular needs of FSWs based on their typology. The paper also highlights and revalidates the efforts of various agencies in response to HIV and promoting safer sexual practices among FSWs, which is reflected in the analysis that the probability of FSWs being approached by system and accessing condoms is very high.

However, the effectiveness of these programmes depends on management planning and implementation that should be participatory in nature—that is, SWs must be involved [active involvement via sex worker collectives etc.]. The paper also emphasized the need to address the issues related to FSWs who are living alone. Living arrangement is an important dimension in response to HIV and access to care. These women should also be brought under the umbrella programmes. ORWs and PEs may play a crucial role in tracing them and providing services.

Nevertheless, the findings of the paper should be interpreted in the light of certain limitations. First, the results identify the probability of a mobile FSW being approached by system and her access to free or social marketed condoms. The results should not be interpreted as correct and consistent use of condoms, as various other factors may attribute to that outcome. Second, the results are not any programme specific, i.e. the increase in the probability is not the result of any particular programmatic or strategic intervention. Hence, it should not be linked to any particular programme and taken as an indicator of the success of that particular programme only. Third, the operation definition of system reach and exposure to intervention is within the sphere of questions asked in the survey of mobile SWs.

## 5. Conclusions

Altogether, the findings do reflect the consistent efforts of government and other international agencies to reach mobile FSWs. Empirical evidences based on the paper highlights that the typology of sex workers is an important attribute in HIV prevention programme (Banandur et al., 2012). Hence, the programmes should be tailored to the specific needs of the sub-groups of this vulnerable population. Most importantly, the paper reflects that the comprehensive TIs are able to reach the most in need among mobile FSWs effectively [e.g. New entrants, highly mobile, reported STIs, tested for HIV ever and serving a high volume of clients].

## Footnotes

**Notes** Note 1. One lakh is equal to 0.1 million

Note 2. A study conducted by Swasti Health Resource Centre & Swathi Mahila Sangha in partnership with Karnataka Health Promotion Trust regarding reaching young, new and high volume sex workers, highlighted that the results have been encouraging and more FSWs were able to access the services provided under the project. http://www.swasti.org/images/projects/thinkpieces/Pragati%20YNH%20approach.pdf

Note 3. Population Council, 2012, “HIV risk/vulnerability among migrant/mobile female sex workers in India”, http://hdl.handle.net/1902.1/20463UNF:5:5KhzdkpKJJxwCGfIa1V0dw==PopulationCouncil[Distributor]V2[Version]
